# Transcriptomic and genomic identification of spliceosomal genes from *Euglena gracilis*: Identification of *Euglena gracilis* spliceosomal genes

**DOI:** 10.3724/abbs.2023143

**Published:** 2023-09-13

**Authors:** Pingwei Gao, Yujie Zhong, Chengfu Sun

**Affiliations:** Scientific Research Center Chengdu Medical College Chengdu 610500 China

**Keywords:** txcavata, spliceosome, intron, transcriptome, *trans*-splicing, SF3B

## Abstract

Diverse splicing types in nuclear and chloroplast genes of protist *Euglena gracilis* have been recognized for decades. However, the splicing machinery responsible for processing nuclear precursor messenger RNA introns, including *trans*-splicing of the 5′ terminal outron and spliced leader (SL) RNA, remains elusive. Here, we identify 166 spliceosomal protein genes and two snRNA genes from *E*. *gracilis* by performing bioinformatics analysis from a combination of next-generation and full-length transcriptomic RNA sequencing (RNAseq) data as well as draft genomic data. With the spliceosomal proteins we identified in hand, the insensitivity of *E*. *gracilis* to some splicing modulators is revealed at the sequence level. The prevalence of SL RNA-mediated *trans*-splicing is estimated to be more than 70% from our full-length RNAseq data. Finally, the splicing proteomes between *E*. *gracilis* and its three evolutionary cousins within the same Excavata group are compared. In conclusion, our study characterizes the spliceosomal components in *E*. *gracilis* and provides the molecular basis for further exploration of underlying splicing mechanisms.

## Introduction

*Euglena gracilis* is a free-living unicellular photosynthetic flagellate that belongs to the Excavata supergroup of eukaryotes [1]. A prominent and unique feature of this organism is its splicing diversity, including both conventional and nonconventional intron splicing of nuclear precursor messenger RNA (pre-mRNA) genes and groups II and III introns splicing of chloroplastic genes [2]. Spliced leader (SL) RNA-mediated *trans*-splicing, the replacement of a 5′ terminal intron sequence (termed outron) of pre-mRNA molecules with a short stretch of noncoding RNA sequence (termed SL exon), is also prevalent in *E*. *gracilis* [ 3, 4] . Additionally, SL-mediated *trans*-splicing has been found in parasitic trypanosomatids within the Excavata supergroup [5].

In *E*. *gracilis*, terminal intronic sequences in pre-mRNA conventional introns, as well as in outrons and SL RNAs, share the same canonical 5′ GT and 3′ AG splicing sites as those in humans and yeast [2]. Therefore, the splicing of these two types of introns is believed to be catalyzed by the spliceosome, a megadalton ribonucleoprotein (RNP) complex. The spliceosome comprises five small nuclear RNAs (snRNAs, U1, U2, U4, U5 and U6) and hundreds of proteins [6]. Spliceosomal proteins can be divided into snRNA-associated proteins (including Sm/LSm core proteins and respective snRNA-related proteins) and non-snRNP proteins (such as ATP helicase Prp2 and its auxiliary factor Spp2) or protein complexes [such as nineteen complex (NTC) and retention and splicing complex (RES)]. The spliceosome catalyzes intron removal in a stepwise manner, consisting of assembly, activation, catalysis and disassembly stages and more than ten consecutive spliceosomal complexes from the A complex to the intron lariat spliceosome (ILS) complex.

Previously, spliceosomal U1 and U5 snRNAs and SL RNA were identified in *E*. *gracilis* [ 7‒ 9] . However, other snRNAs or spliceosomal proteins in *E*. *gracilis* remain unknown. Additionally, a partial genome draft of this organism has recently been reported [10]. Due to the lack of a complete genome sequence, genomic annotation of all spliceosomal genes in *E*. *gracilis* is unavailable. To this end, alternative transcriptomic analysis could be the appropriate choice.

Herein, we identified the spliceosomal proteins and U2 and U6 snRNAs in *E*. *gracilis* from a combination of transcriptomic (next-generation and full-length RNAseqs) and genomic data. Furthermore, the splicing proteomes of *E*. *gracilis* and three other species, including *Trypanosoma brucei*, *Leishmania major* and *Diplonema papillatum*, within Excavata were compared.

## Materials and Methods

### Cell culture and reagents

*E*. *gracilis* cells (strain FACHB-848) were purchased from the Freshwater Algae Culture Collection at the Institute of Hydrobiology (Wuhan, China) and cultured in HUT medium according to the manufacturer’s instructions.

The splicing modulators pladienolide B (PB) and FR901464 were purchased from Adooq Bioscience (Irvine, USA). Herboxidiene (GEX1A) was purchased from Cayman (Ann Arbor, USA), and OTS964 was purchased from TargetMol (Boston, USA). All splicing modulators were dissolved in dimethyl sulfoxide (DMSO) and added to *E*. *gracilis* cells at appropriate concentrations. Cell density was measured with an Ultrospec 3100 pro spectrophotometer (Amersham Biosciences, Marlborough, USA), and cell morphology was examined under a BX63 microscope (Olympus, Tokyo, Japan).

### PCR analysis

Total RNA was extracted using Trizol reagent (Sangon, Shanghai, China), and reverse transcription was performed using a FastKing RT kit with gDNase (Tiangen, Beijing, China). Genomic DNA was extracted from *E*. *gracilis* cells using phenol-chloroform DNA extraction and ethanol precipitation. PCR for selected splicing genes was carried out using Phanta Max Super-Fidelity DNA Polymerase (Vazyme, Nanjing, China) with the primers listed in Supplementary Table S1. DNA sequencing of PCR products was conducted by Tsingke (Beijing, China).

### Full-length transcriptomic RNAseq

Total RNA was extracted from approximately 1×10 ^8^ *E*. *gracilis* cells, and poly(A) mRNA was enriched with oligo(dT) magnetic beads. An Iso-Seq library was prepared according to the isoform sequencing protocol using the Clontech SMARTer PCR cDNA Synthesis kit (Takara, Otsu, Japan) and the BluePippin Size Selection System protocol described by Pacific Biosciences (PN 100-092-800-03; Menlo Park, USA). Subsequently, full-length transcriptomic RNAseq was performed on the PacBio Sequel platform (Pacific Biosciences) by Novogene (Beijing, China). Additionally, to correct any mutations introduced during Iso-seq, a next-generation transcriptomic RNA-seq was simultaneously conducted, and the output data were integrated with the above full-length transcriptomic RNAseq data.

### Data processing

Next-generation transcriptomic RNA-seq data for *E*. *gracilis* (SRR2094880 [4], SRR2628535 [10], and DRR110356-9) and *D*. *papillatum* (SRR21741415) were downloaded from the SRA database ( www.ncbi.nlm.nih.gov/SRA). These SRA files were first converted to the fastq format using the fastq-dump command; sequencing adapters were removed using the Trimmomatic program. Output files of the above *E*. *gracilis* SRA data were combined with our next-generation transcriptomic RNA-seq data. Subsequently, the RNA data for *E*. *gracilis* and *D*. *papillatum* were both applied to the Trinity program suite [11] with default parameters for *de novo* sequence assembly.

For Iso-seq, sequence data were processed using SMRTlink 5.0 software. A circular consensus sequence (CCS) was generated from subread BAM files and classified into full-length and non-full-length reads. Non-full-length and full-length fasta files produced were then fed into the clustering step, which performs isoform-level clustering (ICE), followed by final Arrow polishing. Errors (low-quality sequences) were corrected with LoRDEC V0.7 software using the RNA-seq data from the same sample.

Genomic data of *E*. *gracilis* were downloaded from NCBI under accession number GCA_900893395.1 [10].

### BLAST analysis

Assembled trinity fasta files were searched against the UniProtKB protein database using the Diamond program [12]. The identified hits then underwent a reciprocal BLASTX search against human spliceosomal proteins retrieved from the National Center for Biotechnology Information (NCBI) website ( www.ncbi.nlm.nih.gov/protein). Hits with E-values <10 ^‒6^ were accepted as human spliceosomal orthologues. Putative sequences for the *E*. *gracilis* and *D*. *papillatum* spliceosomal genes were manually inspected by blasting against human spliceosomal proteins, and their open reading frames were subsequently annotated. Human spliceosomal proteins were used as input for querying protein homologues in *T*. *brucei* and *L*. *major* with the BLASTP program and BLOSUM62 matrix [13] with the same standard as above.

### Sequence alignment and structural visualization

Protein sequences were aligned by MEGA software44 with the built-in ClustalW program and visualized by ESPript3 [14]. 3D structures of human SF3B1/PHF5A were extracted from PDB code 6EN4 [15] and CDK11 from 7UKZ [16]. The structure display was made with the UCSF Chimera program [17].

## Results

### Summary of spliceosomal proteins

**Figure 1:**
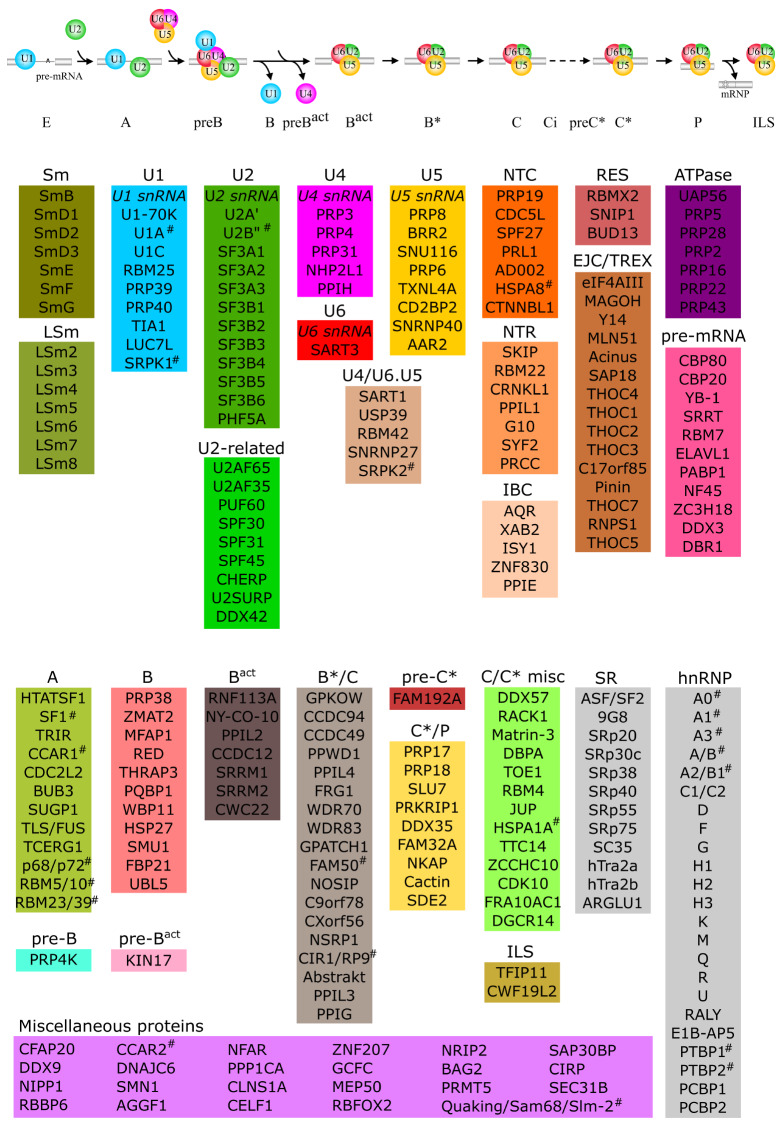
Figure 1 Spliceosomal proteins collected for this work The splicing pathway including thirteen spliceosomal complexes is depicted in the upper panel. Asterisks show the 12 groups of spliceosomal paralogues (U1A/U2B″, SRPK1/SRPK2, HSPA8/HSPA1A, SF1/Quaking/Sam68/Sam-2, CCAR1/CCAR2, p68/p72, RBM5/RBM10, RBM23/RBM39, FAM50A/FAM50B, CIR1/RP9, hnRNP A0/A1/A3/A/B/A2/B1 and PTBP1/PTBP2).

Based on early proteomic and recent structural studies of various purified human spliceosomal complexes [ 18‒ 21] , a complete list of 257 spliceosomal proteins was generated ( Figure 1). This contains: (i) 110 small nuclear ribonucleoprotein (snRNP) and non-snRNP proteins [14 Sm/LSm, 9 U1, 21 U2 and related, 19 U4/U5/U6, 19 nineteen complex (NTC)/NTC-related (NTR)/intron-binding complex (IBC), three retention and splicing complex (RES), 15 exon junction complex (EJC)/transcription and export complex (TREX), 7 ATPase, two cap binding complex (CBC), and one lariat debranching enzyme (LDE)]; (ii) 79 stage-specific proteins [15 A, one pre-B, 11 B, one pre-B ^act^, seven B ^act^, 19 B*/C, one pre-C*, nine C*/P, 13 C/C* misc, and two intron lariat spliceosome (ILS)]; (iii) 43 SR/hnRNP/pre-mRNA-binding proteins (12 SR, 23 hnRNP, and 8 pre-mRNA binding proteins); and (iv) 25 miscellaneous proteins. Typically, 12 sets of these 257 spliceosomal proteins are respective paralogues, including U2B/U2B″, SRPK1/SRPK2, HSPA8/HSPA1A, SF1/Quaking/Sam68/Sam-2, CCAR1/CCAR2, p68/p72, RBM5/RBM10, RBM23/RBM39, FAM50A/FAM50B, CIR1/RP9, hnRNP A0/A1/A3/A/B/A2/B1 and PTBP1/PTBP2, and each set was considered as one protein. Additionally, four sets of proteins, CDC2L2/CDC2L1, TOE1/PNLDC1, CELF1/CELF2 and RBFOX2/RBFOX1, are respective paralogues, with only one protein in each set existing in the above protein list. In total, 241 spliceosomal proteins were used for subsequent bioinformatics analysis ( Supplementary Table S2).

### Identification of *E*. *gracilis* spliceosomal protein genes from next-generation transcriptomic RNAseq

**Figure 2:**
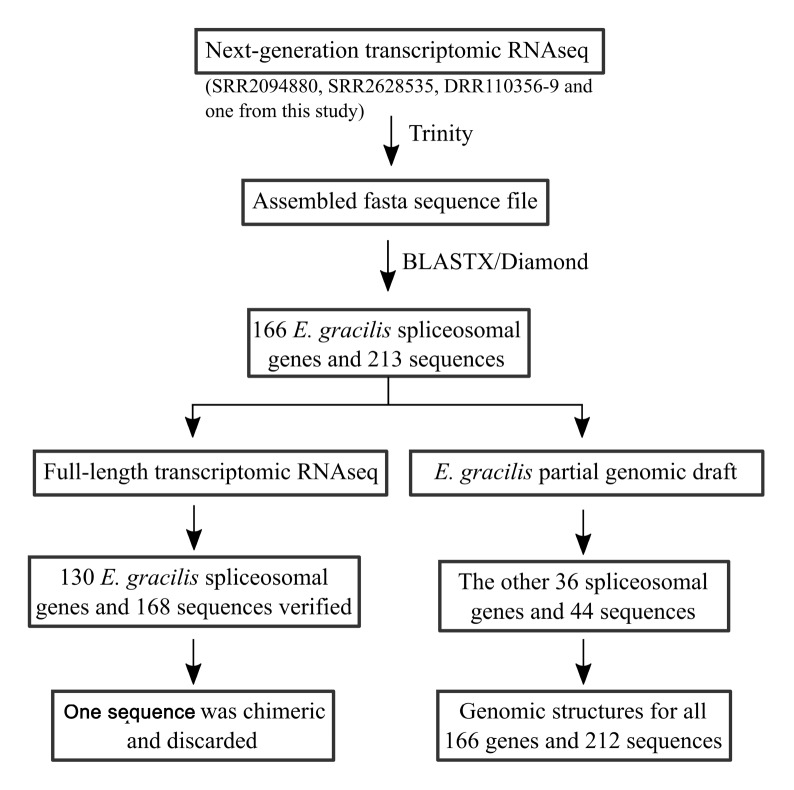
Figure 2 Working procedure for transcriptomic and genomic identification of *E*. *gracilis* spliceosomal protein genes We identified a total of 166 spliceosomal protein genes in E. gracilis ( Supplementary Table S2). We found all snRNP proteins except three (Prp39 of U1, Prp24 of U6, and snRNP27 of U4/U6/U5 snRNP) and all ATP helicases are present in E. gracilis. Except two (PRCC of NTR and CCDC16 of IBC), the NTC/NTR/IBC/RES complexes are also conserved. Notably, large proteins, such as Prp8 and Brr2, could be assembled in full-length, suggesting that the sequencing depth of the RNAseq data we used is well qualified for this analysis. Compared to human spliceosome proteins, most missing proteins in E. gracilis are from SR and related, hnRNP, miscellaneous, B*/C specific, and EJC/TREX proteins. Additionally, we noticed that there are some gene duplications in E. gracilis, including two copies of 10 proteins (SmD3, PUF60, CD2BP2, NY-CO-10, PPIL2, PPIL3, DDX57, PRP43, E1B-AP5 and PPP1CA) and even more copies of seven other proteins (p68/p72, U2AF35, HSPA8/HSPA1A, PRP22, PABP1, DDX3 and PTBP1/PTBP2). A total of 213 sequences of these 166 E. gracilis spliceosomal protein genes were identified in next-generation RNAseq. The presence of multiple copies of the above 17 and the lack of 76 spliceosomal protein genes indicate that the E. gracilis spliceosome may have different features during the splicing process. We concluded that E. gracilis harbors a majority of spliceosomal protein genes, most of which are conserved compared to their human counterparts.

To identify putative spliceosomal proteins in *E*. *gracilis*, we first searched available RNA-seq data deposited in the NCBI Sequence Read Archive (SRA) database. We downloaded three SRA data sets (SRR2094880 [4], SRR2628535 [10], and DRR110356-9) for our analysis. Additionally, as mentioned below, we also performed a next-generation transcriptomic RNAseq analysis. Although this analysis aims to facilitate the processing of full-length transcriptomic RNAseq, we combined the raw data from this analysis with the three SRA data presented above. All data were combined into two files (containing forward and reverse reads) and applied to the Trinity program for sequence assembly. The output of Trinity was searched with BLASTX against the UniProtKB protein database, as mentioned in Materials and Methods. Subsequently, hits of these assembled transcripts were further subject to BLASTX against the corresponding human spliceosomal proteins ( Figure 2). This reciprocal BLAST was previously utilized to identify spliceosomal proteins in other species [ 22, 23] .

### Verification of *E*. *gracilis* spliceosomal genes from full-length transcriptomic RNAseq

To confirm the presence and intactness of the above assembled *E*. *gracilis* splicing protein genes, we further performed full-length transcriptomic RNAseq ( Figure 2). We obtained a total of 258,944 CCS reads, which were further processed into 23,2051 full-length non-chimeric (FLNC) reads, suggesting that most reads (89.6%) passed the Classify program. However, after clustering by hierarchical n*log(n), correcting with next-generation RNAseq by Arrow, and deredundancy by CD-HIT, only 21,850 transcripts and 9669 genes remained. Therefore, we conducted a bioinformatic search for the *E*. *gracilis* spliceosomal proteins mainly based on the original FLNC and CCS reads.

The above 166 spliceosomal protein genes and 213 sequences were subject to BLAST analysis against the full-length transcriptomic RNAseq FLNC and CCS reads. Reads for 130 of these 166 spliceosomal protein genes and 168 of the 213 sequences were identified. In contrast, only 72 spliceosomal protein genes and 98 sequences aligned to the final 21,850 transcripts after CD-HIT treatment. Possible reasons for the missing 36 spliceosomal protein genes in full-length RNAseq compared to next-generation RNAseq include the following: (i) assembled spliceosomal protein genes do not exist but are misaligned, and (ii) the expression level of these proteins is too low to be detected by full-length RNAseq. Indeed, we found both cases for these two reasons. For example, two NY-CO-10 homologues, NY-CO-10A and NY-CO-10B, were identified in next-generation RNAseq. However, the sequence of NY-CO-10A was partially aligned with the sequences in the full-length RNAseq CCS reads. These aligned sequences showed more similarity to human PPID, indicating that the chimeric NY-CO-10A transcript was misassembled during Trinity analysis (consequently, NY-CO-10B was renamed NY-CO-10). Several LSm proteins (LSm2/5/6/7/8) and SF3 proteins (SF3A1/2/SF3B5/6) were absent in CCS reads. However, as mentioned below, genomic sequences of these proteins exist in the partial genome draft [10], indicating that these proteins have low expression and may be undetectable by our full-length RNAseq.

With full-length RNAseq transcripts of most *E*. *gracilis* spliceosomal protein genes in hand, we compared the intactness of these genes between the two types of RNAseq. Of the 168 sequences, the number of (i) intact ORFs found in both RNAseqs, (ii) only next-generation RNAseq or (iii) full-length RNAseq was 118, 40 and 2, respectively. Only nine of these 168 sequences had no intact ORFs in both RNAseqs. Furthermore, we noticed that one of these nine sequences, DDX3B, could generate an intact ORF by assembling sequences from both RNAseqs. This analysis showed that next-generation RNAseq is better than full-length RNAseq in protein identification (158 versus 120). However, we found 16 intact spliceosomal sequences assembled from next-generation RNAseq with the help of full-length RNAseq, indicating that the latter aids in the assembly of sequences for the former.

### Genomic structure of *E*. *gracilis* spliceosomal protein genes

**Figure 3:**
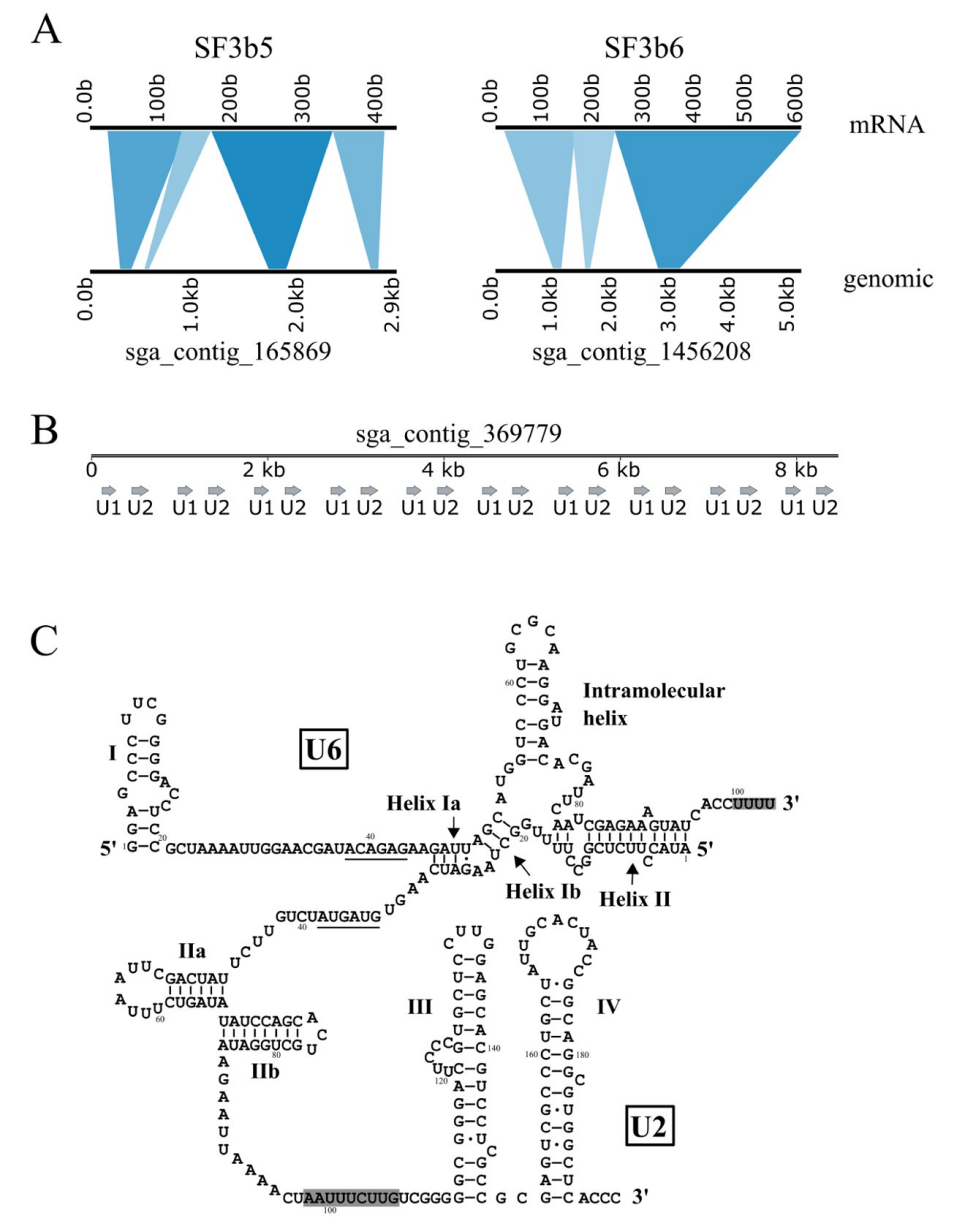
Figure 3 Genomic identification of *E*. *gracilis* spliceosomal protein genes SF3B5 and SF3B6 and snRNA genes U2 and U6 (A) Genomic alignments of SF3B5 and SF3B6. mRNA sequences of SF3B5 and SF3B6 were projected to the corresponding genomic contigs visualized by kablammo [24]. (B) Genomic organization of E. gracilis U1 and U2 in the genomic contig sga_contig_369779. (C) Secondary structure of E. gracilis U2 and U6. Putative RNA motifs are indicated as boxed (Sm binding site of U2 and LSm binding site of U6), underlined (branch point interaction region of U2 and 5′ splice site interaction region of U6), or with arrows (U2/U6 helix Ia, Ib and II).

As mentioned above, 36 of the 166 *E*. *gracilis* spliceosomal protein genes can only be found in next-generation RNAseq. The authenticity of these proteins remains uncertain due to possible assembly errors in the Trinity process. To this end, we searched against the published *E*. *gracilis* genome [10], which is only a partial draft, using the 36 protein sequences described above as input. Fortunately, we found corresponding contigs in the genome sequence for all 36 proteins, suggesting that these proteins exist in *E*. *gracilis* to a large extent. Additionally, we found that for 11 of these 36 proteins, more than half of each protein sequence region could project to respective single genomic contigs. Notably, there are two genomic contigs (sga_contig_165869 and sga_ contig_1456208) that contain the intact sequences (except the SL exon, which is added by *trans*-splicing) of SF3B5 and SF3B6, respectively ( Figure 3A). Additionally, we noticed that the maximal number of exons in a single genomic contig was 8, which was found in sga_contig_217594 for PABP1B. There are also seven exons in single contigs for PRP8, CWC22, RNF113A and HSPA-1.

### Identification of *E*. *gracilis* U2 and U6

Of the five spliceosomal snRNAs, only U1 and U5 had been previously identified in *E*. *gracilis*. Following the above identification of spliceosomal protein genes, we continued to examine the existence of snRNAs in the draft genome [10]. Using previously identified U2 and U6 sequences from *Entosiphon sulcatum* [25], a distant relative of *E*. *gracilis* within the Euglenoids group, as input, we searched putative sequences in the *E*. *gracilis* genome with BLAST. We found an approximate 200-base fragment with high similarity to *E*. *sulcatum* U2 in four contigs (sga_contig_369779, sga_contig_1198033, sga_contig_1446590 and sga_ contig_1602265). We annotated this fragment as the putative *E*. *gracilis* U2. In particular, we noticed ten copies of U2 in tandem in the 8 kb contig sga_contig_369779 ( Figure 3B). After further scrutiny of this contig, we found that U1 is also in this contig with the same tandem organization as U2 ( Figure 3B). Searching for putative U6 sequences with *E*. *sulcatum* U6 failed and neither with U6 from *T*. *brucei* [26]. Therefore, we tried with human U6 as input and identified a fragment of approximately 100 bases in length. This putative *E*. *gracilis* U6 shows, surprisingly, high similarity to human U6 in the internal 58 bases but is more divergent from *E*. *sulcatum* and *T*. *brucei* U6s. We further identified putative domains and motifs in *E*. *gracilis* U2 and U6 and delineated the secondary structure for these two snRNAs ( Figure 3C).

### PCR verification of *E*. *gracilis* spliceosomal genes

**Figure 4:**
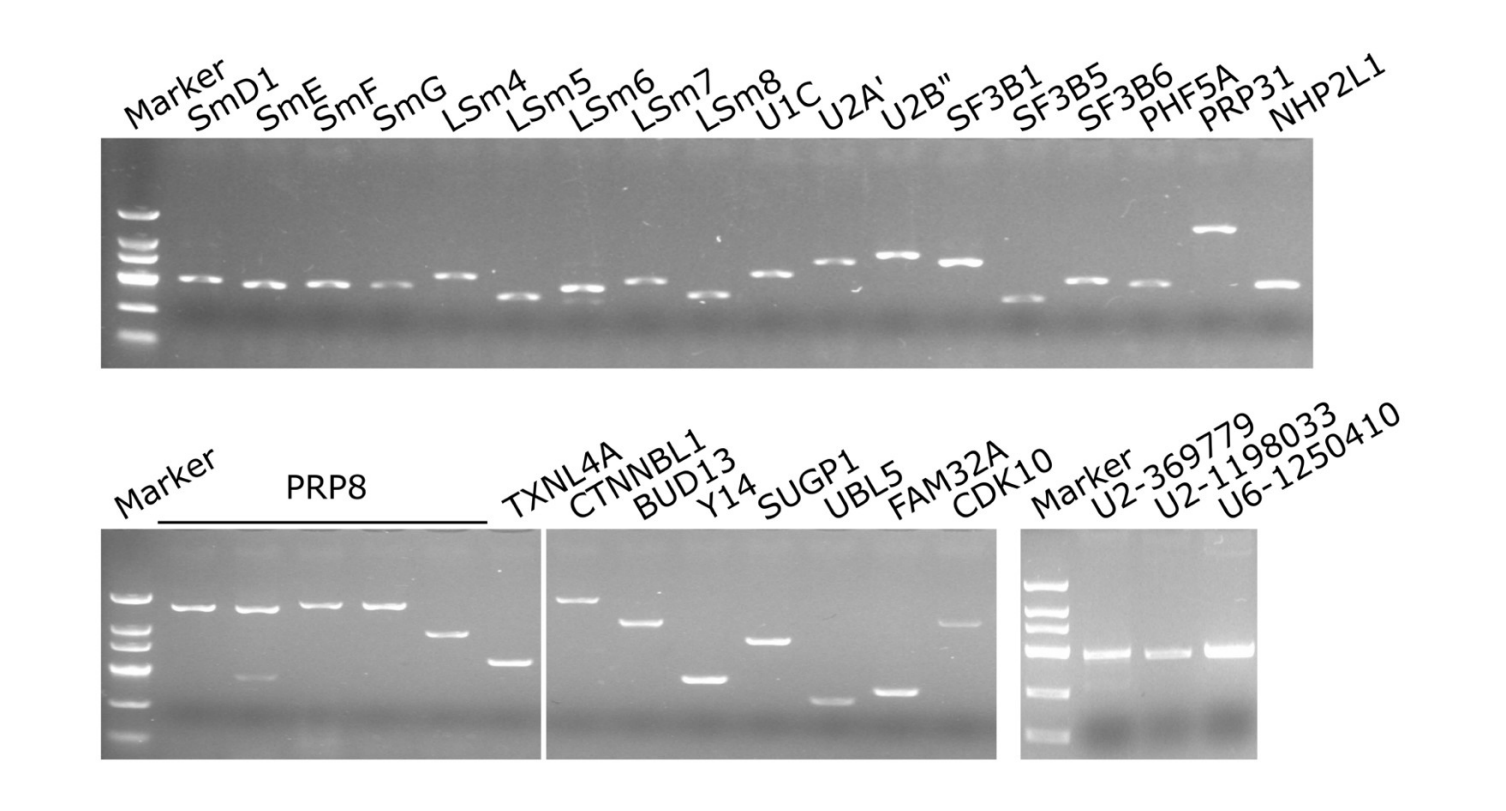
Figure 4 PCR verification of 27 spliceosomal proteins and two snRNA genes Spliceosomal genes were amplified with the respective primers listed in Supplementary Table S1. Numbers after U2 and U6 are genomic contig numbers according to GCA_900893395.1.

We further verified the above identified spliceosomal protein genes with RT-PCR and genomic U2 and U6 snRNA genes with PCR. We first designed primers for 7.3-kb Prp8, the largest protein gene in the spliceosome. However, amplification of full-length Prp8 was unsuccessful. Alternatively, five pairs of primers were designed to amplify different fragments of Prp8. As shown in Figure 4, PCR fragments from 946 bp to 1676 bp in size could be amplified and confirmed to be *E*. *gracilis* Prp8 by DNA sequencing. We further examined other spliceosomal protein genes by RT-PCR, including nine Sm/LSm, ten snRNA specific, three non-snRNP, and four stage-specific genes. DNA sequencing of all these PCR products indicated that all are in agreement with the respective predicted spliceosomal genes. To examine the presence of *E*. *gracilis* U2 and U6 snRNAs, primers were designed according to the genomic sequences of U2 (sga_contig_369779 and sga_contig_1198033) and U6 (sga_contig_1250410). DNA sequencing of amplified PCR fragments also confirmed the presence of these two snRNA species ( Figure 4).

### Prevalence of *trans*-splicing in *E*. *gracilis* genes

A prominent feature of *E*. *gracilis* is *trans*-splicing. Indeed, while identifying spliceosomal protein genes, we noticed the SL exon on many of these genes. After combining the transcriptomic and genomic data above, we obtained 166 spliceosomal protein genes and 212 sequences in *E*. *gracilis*. Of these 212 sequences, we found the SL exon in 193 sequences, accounting for 91%. Additionally, we checked the ratio of *trans*-splicing in our full-length RNAseq. Of the final 9669 genes, 7028 have SL exons, representing 72.7%. These ratios suggest that most *E*. *gracilis* genes, especially spliceosomal protein genes, are subject to *trans*-splicing.

### Molecular basis for the inability of splicing modulators in *E*. *gracilis*

**Figure 5:**
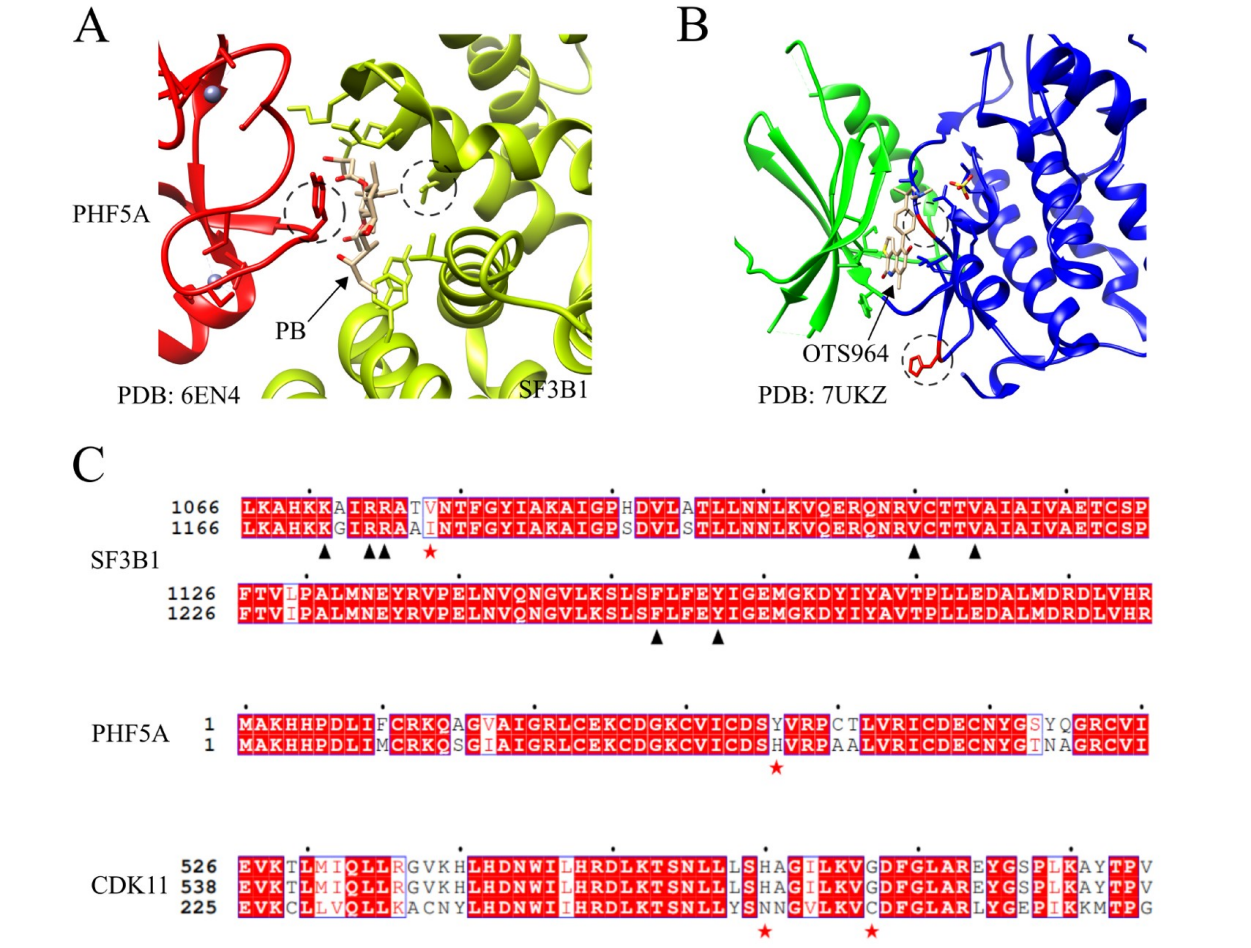
Figure 5 Sequence comparison of SF3B1/PHF5A between humans and *E*. *gracilis* (A) Structure of human SF3B1/PFH5A with PB. PHF5A is colored red, and SF3B1 is colored olive. SF3B1 Val 1078 and PHF5A Cys 36 are labeled with dashed circles. PB, which is harbored between SF3B1 and PHF5A, is indicated with an arrow. (B) Structure of human CDK11B with OTS964. OTS964 is indicated with an arrow. CDK11B domains that contain OTS964 are colored green and blue. His 572 and Gly 579 are labeled with dashed circles. (C) Sequence alignments of human and E. gracilis SF3B1, PHF5A and CDK11. For each alignment, the upper sequence(s) were from human proteins, and the lower sequence was from E. gracilis. Residues that influence the interaction with PB or OTS964 are indicated with red stars. Other residues in SF3B1 that interact with PB are indicated with dark triangles. The alignments were generated by ESPript [14].

Many identified splicing modulators can intervene in human splicing by acting on specific spliceosomal proteins, such as PB and GEX1A. However, PB and several other splicing modulators have been found to have no effect on *E*. *gracilis* ( Supplementary Figure S1), which is consistent with previous report [27]. As PB acts on the SF3B1/PHF5A complex [15] ( Figure 5A), we compared these proteins between humans and *E*. *gracilis*. The overall sequence similarity of SF3B1 is 79%, and of PHF5A is 92%. Alignment of both proteins between the two species indicated that of the eight residues of the human SF3B1 protein that can interact with PB, Val ^1078^ is changed to Ile in *E*. *gracilis* ( Figure 5C). The human PHF5A Cys ^36^ is also changed to His in *E*. *gracilis* ( Figure 5C). As mutations in these two residues previously showed an alleviated effect of PB on SF3b binding [ 15, 28] , we suggested that *E*. *gracilis* may be insensitive to splicing modulators such as PB that targets SF3b ( Supplementary Figure S1).

Furthermore, our findings revealed that OTS964, which targets human spliceosomal CDK11B/CDC2L1 [ 16, 29, 30] ( Figure 5B), does not affect *E*. *gracilis* ( Supplementary Figure S1). In *E*. *gracilis*, we identified only one protein that shows similarity (approximately 70%) to the C-terminal kinase domain of both CDK11 paralogues (CDK11A and CDK11B). We compared the sequences of all three proteins. Indeed, the two residues that maintain OTS964 activity on human CDK11, His ^572^ and Gly ^579^, are all mutated to other residues in the putative *E*. *gracilis* CDK11 ( Figure 5C). From the above analysis, spliceosomal proteins in *E*. *gracilis* show minor but crucial divergence from those of humans and render splicing modulators insensitive in this organism.

### Comparison of the splicing proteomes between *E*. *gracilis* and other Excavate species

Free-living *E*. *gracilis* and parasitic *T*. *brucei* and *L*. *major* belong to the Excavate group and have widespread *trans*-splicing. During snRNA identification, searching the *E*. *gracilis* genome with snRNAs from *T*. *brucei* failed. Divergence of snRNA between *E*. *gracilis* and *T*. *brucei* encouraged us to explore the splicing difference between these two Excavate species. To this end, we searched *T*. *brucei* and *L*. *major* for 241 spliceosomal proteins using the BLASTP program. We also included previously identified mass spectrometric results [31]. Typically, 78 spliceosomal protein genes in *T*. *brucei* and 76 in *L*. *major* were identified, much less than those in *E*. *gracilis* ( Supplementary Table S2). Only very conserved snRNP proteins, including Sm/LSm, U2, U4/U6, U5, and ATP helicase proteins, remain in these two parasitic species. Comparison between *T*. *brucei* and *L*. *major* indicated that there are only very slight differences in spliceosomal protein composition, including SRSF7, hnRNP D and hnRNP F/H only in *T*. *brucei* and Y14 only in *L*. *major*.

The considerable differences in spliceosomal protein composition between *E. gracilis* and its parasitic cousins prompted us to examine more species in the Excavata group. To this end, we selected *D*. *papillatum*, a marine euglenozoan protist. In total, we identified 121 spliceosomal proteins in *D*. *papillatum*, the number of which sits between those of *E*. *gracilis* and parasitic kinetoplastids. In addition, we noticed that only four proteins are present in Excavate species other than *E*. *gracilis*, including ELAV1 and hnRNP D/F/H. In summary, our findings indicated that *E*. *gracilis* and parasitic *T*. *brucei* and *L*. *major* show a clear difference in spliceosomal composition, presumably indicative of their respective free-living and parasitic styles.

## Discussion

This research identified the spliceosomal genes in *E*. *gracilis*, including 212 spliceosomal protein sequences and 2 snRNAs. We first determined more than 200 spliceosomal proteins and then identified 166 from the next-generation transcriptomic RNAseq of *E*. *gracilis*. These spliceosomal protein genes were further verified with full-length transcriptomic RNAseq and a partial genomic draft.

Nuclear pre-mRNAs of *E*. *gracilis* contain many introns, including the 5′ terminal outrons and internal conventional and nonconventional introns. Outrons are spliced with SL RNA by *trans*-splicing. Both *trans*-splicing and conventional intron *cis*-splicing occur on the spliceosome. The spliceosomal protein genes in *E*. *gracilis* identified here suggest that most snRNP and related proteins, as well as non-snRNP NTC/NTR/IBC/RES complexes, are conserved in this organism. In comparison, most catalytic stage-specific proteins and pre-mRNA binding/SR/hnRNP proteins are lost. Previously, Ebenezer *et al*. [10] identified some spliceosomal proteins in *E*. *gracilis* through assembled next-generation RNAseq data. However, compared to our analysis, their annotation was incomplete, presumably due to the relatively small size of their RNAseq, and contained some misassignments ( *e*. *g*., Prp11/SF3A2 they identified is actually SF3A3).

In accordance with the more than one hundred spliceosomal proteins identified here, *E*. *gracilis* is believed to contain all five snRNAs. Together with U2 and U6 identified here, sequences of four snRNAs were available, and only U4 remains unknown. Of the four snRNAs, U1, U2, and U5 are arranged all in tandem in the genome. The tandem genomic organization also holds true for SL RNA [7]. Therefore, it is conceivable that *E*. *gracilis* demands high levels of these snRNA/SL RNAs for the splicing of pre-mRNA genes during gene expression, which tandem organization favors. Breckenridge *et al*. [8] immunoprecipitated several species of *E*. *gracilis* snRNA. In addition to U1, they donated two other bands to U2 and U4. However, the size of their U2 seems smaller than what we identified here. Therefore, it is unclear whether there is a short version of U2 in *E*. *gracilis*. With the availability of spliceosomal proteins and four snRNAs, especially catalytic U2, U5, and U6, it is, in principle, possible to construct the 3D structure of an *E*. *gracilis* spliceosome. However, as seen in *T*. *brucei* [31], there may exist some specific splicing proteins in *E*. *gracilis* as well. Therefore, experimental purification of the spliceosomal complexes and composition identification are required to provide a complete understanding of the splicing machinery in this organism. Additionally, it is necessary to experimentally verify the spliceosomal protein and snRNA components we identified here.

The prevalence of SL-mediated *trans*-splicing has been previously estimated to be approximately 50% in *E*. *gracilis* genes [ 3, 4] . Here, we evaluated the ratio of *trans*-splicing based on our full-length transcriptomic RNAseq and found it to be slightly higher than previous estimations. Although *trans*-splicing in *E*. *gracilis* is prevalent, the significance of this type of splicing remains unknown in this organism. Unlike *T*. *brucei* and *L*. *major*, whose *trans*-splicing benefits their parasitic lifestyles, *E*. *gracilis* is free-living and contains chloroplasts. We speculate that adding an SL exon to the 5′ termini of most genes may help *E*. *gracilis* control the activity of these genes for export and translation. Additionally, the identification of spliceosomal genes in *E*. *gracilis* provides no clue to the molecular process of *trans*-splicing, which requires further experimental investigation.

Although *E*. *gracilis* harbors most of the spliceosomal proteins compared to humans, the evolutionary distance between these two species has deposited high sequence divergence in the splicing system. Accordingly, most splicing modulators examined thus far are ineffective for *E*. *gracilis*. Therefore, to investigate the splicing mechanism in this organism, new splicing modulators need to be developed either by *in vitro* virtual screening or by experimental characterization.

## Supplementary Data

Supplementary data is available at *Acta Biochimica et Biophysica Sinica* online.

## Data Availability

*E*. *gracilis* RNA-seq transcriptomic data are available from the NCBI SRA database under the project accession number PRJNA913467. Annotated *E*. *gracilis* and *D*. *papillatum* spliceosomal gene sequences have been deposited in GenBank.

## Supporting information

SupplementaryFig

Supplementary_T1

Supplementary_T2
