# Supplementary material for: Transcriptomic and genomic identification of spliceosomal genes from Euglena gracilis : Identification of Euglena gracilis spliceosomal genes
Source: Acta Biochim Biophys Sin (Shanghai). 2023 Sep 13;55(11):1740–8. doi: 10.3724/abbs.2023143 (PMC10679874; doi:10.3724/abbs.2023143)
Supplement: SupplementaryFig [file SupplementaryFig.pdf]

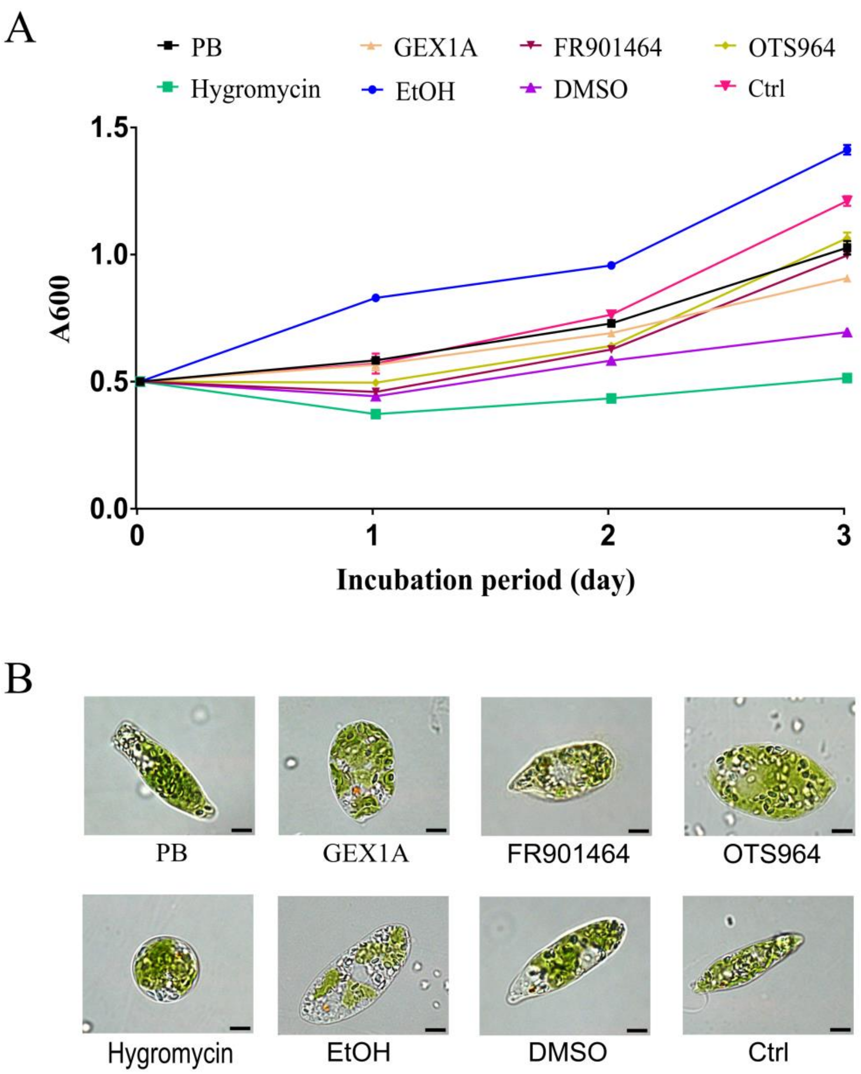

Supplementary Figure S1. Effect of splicing modulators on *E. gracilis* growth and morphology (A) Growth of *E. gracilis* treated with different splicing modulators. Splicing modulators including PB (final concentration 1 µmol), GEX1A (1 µmol), FR901464 (20 µmol) and OTS964 (10 µmol) were added to *E. gracilis* cells and cell density was measured daily for three days. EtOH, which promotes growth of *E. gracilis* cells, was used a positive control and added at a ratio of 1:1000. Hygromycin (50 µg/mL) inhibits *E. gracilis* cell growth and was used as a negative control. DMSO was added at a ratio of 1:100. (B) Cell morphology of *E. gracilis* after treatment with different splicing modulators for three days. The micrographs were taken at 100× magnification. Scale bar: 10 µm.
