# Supplementary material for: Transcriptomic and genomic identification of spliceosomal genes from Euglena gracilis : Identification of Euglena gracilis spliceosomal genes
Source: Acta Biochim Biophys Sin (Shanghai). 2023 Sep 13;55(11):1740–8. doi: 10.3724/abbs.2023143 (PMC10679874; doi:10.3724/abbs.2023143)
Supplement: Supplementary_T1 [file Supplementary_T1.pdf]

Supplementary Table S1.Sequences of primers used in this work

Primer name	Primer sequence (5′ →3′)	PCR product length (bp)
egSmD1_F	GTGTTCAATGAAGCTCGTAAG	482
egSmD1_R	GCAGTCAAGAGCCGCCACC	
egSmE_F	TGGTTGGGCAAACAGCAGC	451
egSmE_R	GCGGGGCATGTACAGGGTG	
egSmF_F	GCTCCACCTATGAGCTTTGAG	468
egSmF_R	TGATTGGGCAGCCAACTATT	
egSmG_F	CAACTCACACAAAAAAATGGC	466
egSmG_R	GCTGCCGCACAAAAAAACC	
egLsm4_F	AGCCCACCCCCATATGCTG	564
egLsm4_R	GAACAGGGCAGGGGATCGG	
egLsm5_F	ACTACCACATGGCTGGCGC	391
egLsm5_R	CAAACGTGGGCAGTGGTGG	
egLsm6_F	GGGCTTCACAATGGCCGAC	461
egLsm6_R	TGTCGTTGAGGAGGAGGGG	
egLSm7_F	CTCAACAAGTAATTGAATGCCTGC	517
egLSm7_R	GTGGAACGACAGCGGAGAA	
egLSm8_F	GCCAGCCATGTCTCAGCAA	402
egLSm8_R	GCCAGTTGGTGGAGGTTGT	
egU1C_F	GGGTCGGTGGGATGCCGA	586
egU1C_R	TGGGGTCGGTCCCAGGCT	
egU2A'_F	TCGAATGCGGTTGACAGC	748
egU2A'_R	TGATGAGCATCCACCACAG	
egU2B"_F	GGGTCTTTTTTGGAGGGCG	844
egU2B"_R	GGGATGTGTACCAGCGAGG	
egSF3B1_F	ACGAGAAGGTGCAAGAGAATTG	749
egSF3B1_R	CTGGTGGCTGATGTATATGTTGT	
egSF3B5_F	ATGGCCGACCCATTCGTC	386
egSF3B5_R	GTGTCACCGTGTCACTGTG	
egSF3B6_F	CCTCCCCCCGCACCATGT	526
egSF3B6_R	CGCCCCAGAGGGTTCACC	
egPHF5A_F	CGCAAACCATGGCAAAGCA	496
egPHF5A_R	ACGACGTCATGCGGCGAGG	
egPrp31_F	CATTATGGCAGAGGCTTTGG	1483
egPrp31_R	GCAAGGAGAGTGAGAAGAGG	
egSnu13_F	AAGTTAGGATCTGCTTCTGGTTCAA	445
egSnu13_R	CGTCATCTGCGTGGCTTCTT	
egPrp8_F	CGGCCAAGATGTCCATTCC	1554
egP8-1.5k_R	CATTGAGCACCCAGCACTTC	
egP8-1.5k_F	GAAGTGCTGGGTGCTCAATG	1545
egP8-3k_R	CAGATGTCCGTCAGGTTGTTG	
egP8-3k_F	CAACAACCTGACGGACATCTG	1676
egP8-4.7k_R	GCTTCTTGTACTTCATGCTCTCC	
egP8-4.7k_F	GGAGAGCATGAAGTACAAGAAGC	1653
egP8-6.3k_R	GGTGAGGTGGTGGTGATGAG	
egP8-6.3k_F	CTCATCACCACCACCTCACC	946
egPrp8_R	GGGGGGGAAAGGTTGAGTATG	
egU5-15K_F	TGCTGCCTCATTTGCACAG	588
egU5-15K_R	GGTCTACAGCTGACCCCCC	
egCTNNBL1_F	CGTGGAGAAGGCGGAGAATGA	1854
egCTNNBL1_R	TCACCGTTGGCTCCCGAAAC	
egBud13_F	AGACCAGCCTGACGATGATGAA	1166
egBud13_R	GCGAGATTTCCTTTGCGATCCA	
egY14.CK_F	TTGACGAGGAAGACGCCACTT	463
egY14.CK_R	GCAATTCGGTGCAGTCTCACAA	
egSUGP1_F	CAGCGTAGAGCGTTCCAGAGTA	858
egSUGP1_R	TTCCGAGGGTCAGACACATCAG	

egUBL5_F	CGATGCCGATGATCGAGGTGA	324
egUBL5_R	TCAGGTAACGGACAGCAAGAGG	
egFAM32A_F	AGCAATGTCATTCCTGGCAAGC	389
egFAM32A_R	GCACCACCTCGTGAGAAACAAG	
egCDK10_F	TGGAAGGAGTTGCTGCTTGCT	1178
egCDK10_R	GGGATGCTCGGCGAAATATGG	
egU2-369779_F	CGTCGGAAGTGTCTGATGGTCT	509
egU2-369779_R	CTGCCGCCGACCTCATAATC	
egU2-1198033_F	ACCAGTGCAGTCTGGATGGC	453
egU2-1198033_R	TGGCTTCGCTTGGTGGCTTC	
egU6-1250410_F	CGGTCTCCAAGTCCTCAGTCAG	496
egU6-1250410_R	CAGTTGCCGTCGGTAGAATGC	
