# Supplementary material for: Transcriptomic and genomic identification of spliceosomal genes from Euglena gracilis : Identification of Euglena gracilis spliceosomal genes
Source: Acta Biochim Biophys Sin (Shanghai). 2023 Sep 13;55(11):1740–8. doi: 10.3724/abbs.2023143 (PMC10679874; doi:10.3724/abbs.2023143)
Supplement: Supplementary_T2 [file Supplementary_T2.pdf]

Supplementary Table S2. Spliceosomal protein genes identified in *E. gracilis* and comparison to *H. sapiens* and three other Euglenozoa species

Complex	Genes	*H. sapiens*	*E. gracilis*[#]	*T. brucei*[$]	*L. major*	*D. papillatum*[#]
Sm	SmB	NP_003082	OP185536	XP_951644	XP_003722006	OQ872397
	SmD1	NP_008869	OP185537	XP_845935	XP_001683257	OQ872398
	SmD2	NP_808210	OP185538	XP_951729	XP_001686081	OQ872399
	SmD3	NP_004166	OP185539<br>OP185540	XP_844239	XP_001686492	OQ872400<br>OQ872401
	SmE	NP_003085	OP185541	XP_845391	XP_001684716	OQ872402
	SmF	NP_003086	OP185542	XP_827316	XP_003722843	OQ872403
	SmG	NP_003087	OP185543	XP_829487	XP_001685423	OQ872404
LSm	LSm2	NP_067000	OP185544			OQ872405
	LSm3	NP_055278	OP185545			OQ872406
	LSm4	NP_036453	OP185546	XP_829452	XP_001685387	OQ872407
	LSm5	NP_036454	OP185547			OQ872408
	LSm6	NP_009011	OP185548			OQ872409
	LSm7	NP_057283	OP185549	XP_845073	XP_001684007	OQ872410
	LSm8	NP_057284	OP185550			OQ872411
U1 snRNP	U1-70K	NP_003080	OP185551	XP_847313	XP_001682242	OQ872412
	U1A[&]	NP_004587	OP185562<br>OP185563	XP_843940	XP_003722162	OQ872413<br>OQ872414
	U1C	NP_003084	OP185552			
	RBM25	NP_067062	OP185553			
	PRP39	NP_060392	No hit			
	PRP40	NP_060362<br>NP_0010268<br>68	OP185554<br>OP185555			OQ872415
	TIA1	NP_071505	OP185556			
	LUC7L	NP_060502	OP185557			
	SRPK1[&]	NP_003128	OP185558<br>OP185559<br>OP185560	XP_845614<br>XP_845720	XP_001684968<br>XP_001684102	OQ872416<br>OQ872417<br>OQ872418<br>OQ872419
U2 snRNP	U2A'	NP_003081	OP185561	XP_827916	XP_001683928	OQ872420
	U2B''[&]	NP_003083	OP185562<br>OP185563	XP_843940	XP_003722162	OQ872413<br>OQ872414
	SF3A1	NP_005868	OP185564	XP_828057	XP_001682710	OQ872421
	SF3A2	NP_009096	OP185565	XP_951536	XP_003721618	OQ872422
	SF3A3	NP_006793	OP185566	XP_845437	XP_001684786	OQ872423
	SF3B1	NP_036565	OP185567	XP_829247	XP_001684544	OQ872424
	SF3B2	NP_006833	OP185568	XP_845321	XP_001684647	OQ872425
	SF3B3	NP_036558	OP185569	XP_846318	XP_001687566	OQ872426
	SF3B4	NP_005841	OP185570	XP_844118	XP_003722112	OQ872427
	SF3B5	NP_112577	OP185571	XP_827364	XP_003722805	OQ872428
	SF3B6	NP_057131	OP185572	XP_822981	XP_001686883	OQ872429
	PHF5A	NP_116147	OP185573	XP_822974	XP_001686871	OQ872430
U2 related	U2AF65	NP_0010124<br>96	OP185574			
	U2AF35	NP_0010203<br>74	OP185575<br>OP185576	XP_822572	XP_003721697	OQ872431

			OP185577			
	PUF60	NP_510965	OQ397584<br>OQ397585			
	SPF30	NP_005862	OP185579			OQ872432
	SPF31	NP_055095	No hit			
	SPF45	NP_116294	OP185580			OQ872433
	CHERP	NP_006378	OP185581			
	U2SURP	NP_0010738<br>84	OP185582	XP_846348	XP_001687538	OQ872434<br>OQ872435
	DDX42	NP_031398	OP185583			OQ872436
U6 snRNP	SART3	NP_055521	No hit			
U4/U6<br>di-snRNP	PRP3	NP_004689	OP185584			OQ872437
	PRP4	NP_004688	OP185585	XP_822354	XP_001683064	OQ872438
	PRP31	NP_056444	OP185586	XP_823290	XP_001685738	OQ872439<br>OQ872440
	NHP2L1	NP_0010037<br>96	OP185587	XP_803820	XP_001682066	OQ872441
	PPIH	NP_006338	OP185588	XP_845254<br>XP_845256<br>XP_845262	XP_001684591	OQ872442
U5 snRNP	PRP8	NP_006436	OP185589	XP_827386	XP_003722790	OQ872443
	BRR2	NP_054733	OP185590	XP_844903	XP_001681794<br>XP_001682350	OQ872444
	SNU116	NP_004238	OP185591	XP_829601	XP_001685543	OQ872445
	PRP6	NP_036601	OP185592			OQ872446
	TXNL4A	NP_006692	OP185593	XP_847089	XP_001683400	OQ872447
	CD2BP2	NP_006101	OP185594<br>OP185595			
	SNRNP40	NP_004805	OP185596			OQ872448
	AAR2	NP_056326	OP185597			
U4/U6.U5<br>tri-nRNP	SART1	NP_005137	OP185598			
	USP39	NP_006581	OP185599			
	RBM42	NP_077297	OP185600			OQ872449
	SNRNP27	NP_006848	No hit			
	SRPK2[&]	NP_872634	OP185558<br>OP185559<br>OP185560	XP_845614<br>XP_845720	XP_001684968<br>XP_001684102	OQ872416<br>OQ872417<br>OQ872418<br>OQ872419
NTC	PRP19	NP_055317	OP185601	XP_951696	XP_003722060	OQ872450
	CDC5L	NP_001244	OP185602	XP_844880	XP_001682333	OQ872451
	SPF27	NP_005863	OP185603			
	PRL1	NP_002660	OP185604	XP_823238	XP_001687067	OQ872452
	AD002	NP_057487	OP185605			OQ872453
	HSPA8[&]	NP_006588	OP185606<br>OP185607<br>OQ397586<br>OQ397587<br>OQ397588<br>OQ397589	XP_829198	XP_001684128<br>XP_001684563<br>XP_001684568<br>XP_001684402	OQ872454<br>OQ872455<br>OQ872456<br>OQ872457
	CTNNBL1	NP_110517	OP185608			OQ872458
NTR	SKIP	NP_036377	OP185609			OQ872459
	RBM22	NP_060517	OP185610			OQ872460

	CRNKL1	NP_057736	OP185611	XP_823189	XP_001687012	OQ872461
	PPIL1	NP_057143	OP185612	XP_827807	XP_001687067	OQ872462<br>OQ872463
	G10	NP_003901	OP185613	XP_822907	XP_001681198	
	SYF2	NP_056299	OP185614			OQ872464
	PRCC	NP_005964	No hit			
IBC	AQR	NP_055506	OP185615			OQ872465
	XAB2	NP_064581	OP185616	XP_844808	XP_001683515	OQ872466
	ISY1	NP_065752	OP185617	XP_847026	XP_001680905	OQ872467
	ZNF830	NP_443089	No hit			
	PPIE	NP_006103	OP185618			OQ872468
RES complex	RBMX2	NP_057108	OP185619	XP_845742	XP_001684080	OQ872469
	SNIP1	NP_078976	OP185620	XP_844126	XP_003722104	OQ872470
	BUD13	NP_116114	OP185621			
EJC/TREX	eIF4AIII	NP_055555	OP185622	XP_828941	XP_001684436	OQ872471
	MAGOH	NP_002361	OP185623	XP_845612	XP_001684966	OQ872472
	Y14	NP_005096	OP185624		XP_001684082	OQ872473
	MLN51	NP_031385	No hit			
	Acinus	NP_055792	No hit			
	SAP18	NP_005861	No hit			OQ872474
	THOC4	NP_005773	No hit			
	THOC1	NP_005122	OP185625			
	THOC2	NP_0010750<br>19	OP185626			
	THOC3	NP_115737	OP185627			OQ872475
	C17orf85	NP_0011075<br>90	No hit			
	Pinin	NP_002678	No hit			
	THOC7	NP_079351	OP185628			
	RNPS1	NP_006702	OP185629			
	THOC5	NP_0010028<br>77	No hit			
A specific	HTATSF1	NP_055315	OP185630			OQ872476
	SF1[&]	NP_004621	OP185631			
	TRIR	NP_076943	No hit			
	CCAR1[&]	NP_060707	OP185632<br>OP185633			
	CDC2L2	NP_076916	OP185634			OQ872477
	BUB3	NP_0010077<br>94	OP185635			
	SUGP1	NP_757386	OP185636			
	TLS/FUS	NP_004951	No hit			
	TCERG1	NP_006697	OP185637			
	p68/p72	NP_004387<br>NP_006377	OQ397590<br>OQ397591<br>OQ397592<br>OQ397593<br>OQ397594<br>OQ397595	XP_822905	XP_001680939<br>XP_001686790	OQ872478

	RBM5/RBM10	NP_005769<br>NP_005667	No hit			
	RBM23/RBM39	NP_001070819<br>NP_004893	OP185641			OQ872479
pre-B specific	PRP4K	NP_003904	OP185642<br>OP185643<br>OP185644			OQ872480
B specific	PRP38	NP_116253<br>NP_060531	OP185645<br>OP185646			OQ872481
	ZMAT2	NP_653324	OP185647			OQ872482
	MFAP1	NP_005917	OP185648			OQ872483
	RED	NP_006074	OP185649			OQ872484
	THRAP3	NP_001308400	No hit			
	PQBP1	NP_001027553	OP185650			
	WBP11	NP_057396	No hit			
	HSP27	NP_001531	No hit			
	SMU1	NP_060695	OP185651			OQ872485
	FBP21	NP_009118	No hit			
	UBL5	NP_077268	OP185652			OQ872486
pre-Bact specific	KIN17	NP_036443	OP185653			OQ872487
Bact specific	RNF113A	NP_008909	OP185654			
	NY-CO-10	NP_005860	OP185655			OQ872488
	PPIL2	NP_055152	OQ397596<br>OQ397597			OQ872489
	CCDC12	NP_653317	OP185657			
	SRRM1	NP_005830	No hit			
	SRRM2	NP_057417	OP185658			OQ872490
	CWC22	NP_001362958	OP185659	XP_829139	XP_001687102	OQ872491
B*/C specific	GPKOW	NP_056513	OP185660			
	CCDC94	NP_060544	OP185661			
	CCDC49	NP_060218	No hit			
	PPWD1	NP_056157	OP185662			OQ872492
	PPIL4	NP_624311	OP185663	XP_845045	XP_001682143	OQ872493
	FRG1	NP_004468	No hit			
	WDR70	NP_060504	OP185664			
	WDR83	NP_115708	No hit			OQ872494
	GPATCH1	NP_060495	OP185665			OQ872495
	FAM50	NP_004690<br>NP_036267	OP185666			
	NOSIP	NP_057037	OP185667			
	C9orf78	NP_057604	No hit			
	CXorf56	NP_071384	No hit			
	NSRP1	NP_115517	No hit			
	CIR1/RP9	NP_004873<br>NP_976033	No hit			

	Abstrakt	NP_057306	OP185668			OQ872496
	PPIL3	NP_570981	OQ397598<br>OQ397599	XP_827430	XP_003722755	OQ872497
	PPIG	NP_004783	No hit			
pre-C*	FAM192A	NP_079222	No hit			
C*/P specific	PRP17	NP_056975	OP185671	XP_843787	XP_001683944	OQ872498
	PRP18	NP_003666	OP185672			OQ872499
	SLU7	NP_006416	OP185673	XP_844974	XP_001681147	OQ872500
	PRKRIP1	NP_078929	No hit			
	DDX35	NP_068750	OP185674			OQ872501
	FAM32A	NP_054796	OP185675			
	NKAP	NP_078804	OP185676	XP_823332	XP_001685799	OQ872502
	Cactin	NP_067054	OP185677	XP_829224	XP_001684517	OQ872503
	SDE2	NP_689821	OP185678			
C/C* misc	DDX57	NP_945314	OQ397600<br>OQ397601	XP_829273	XP_001681170	
	RACK1	NP_006089	OP185680	XP_829201	XP_001684560	OQ872504<br>OQ872505
	Matrin-3	NP_0011818<br>83	No hit			
	DBPA	NP_003642	OP185682			
	TOE1	NP_079353	No hit			
	RBM4	NP_002887	OP185683<br>OQ397602	XP_951655	XP_003722019	
	JUP	NP_0013397<br>02	No hit			
	HSPA1A[&]	NP_005336	OP185606<br>OP185607<br>OQ397586<br>OQ397587<br>OQ397588<br>OQ397589	XP_829198	XP_001684563<br>XP_001684568	
	TTC14	NP_597719	OP185684			
	ZCCHC10	NP_0012877<br>45	OP185685			
	CDK10	NP_443714	OP185686			OQ872506
	FRA10AC1	NP_0013346<br>41	OP185687			
	DGCR14	NP_073210	OP185688			OQ872507
ILS specific	TFIP11	NP_0010086<br>97	OP185689			
	CWF19L2	NP_689647	No hit			
ATP helicase	UAP56	NP_004631	OP185690	XP_822312	XP_001683110	OQ872508
	PRP5	NP_055644	OP185691	XP_845030	XP_001681037	OQ872509
	PRP28	NP_004809	OP185692			OQ872510
	PRP2	NP_003578	OP185693	XP_822773	XP_001686659	OQ872511
	PRP16	NP_054722	OP185694	XP_828648	XP_001683625	OQ872512
	PRP22	NP_004932	OQ397603<br>OQ397604<br>OQ397605<br>OQ397606	XP_822963<br>XP_823137	XP_001686861	OQ872513<br>OQ872514
	PRP43	NP_001349	OQ397607<br>OQ397608	XP_844789<br>XP_844389	XP_003722513	OQ872515
pre-mRNA/intr	CBP80	NP_002477	OP185697			

on binding proteins	CBP20	NP_031388	OP185698	XP_845318	XP_001684644	OQ918096
	YBX1	NP_004550	OP185682			
	SRRT/ASR 2B	NP_056992	No hit			
	RBM7	NP_057174	No hit			
	ELAVL1	NP_001410	No hit	XP_847495	XP_001683802<br>XP_001683803	OQ872516
	PABP1	NP_002559	OQ397609<br>OQ397610<br>OQ397611<br>OQ397612<br>OQ397613	XP_843988<br>XP_843985<br>XP_827237<br>XP_827358	XP_003722897	OQ872517<br>OQ872518<br>OQ872519<br>OQ872520
	NF45	NP_004506	No hit			
	ZC3H18	NP_653205	No hit			
	DDX3	NP_001347	OQ397614<br>OQ397615<br>OQ397616<br>OQ397617<br>OQ397618	XP_827934<br>XP_827494	XP_001685353	
	Dbr1	NP_057300	OP185699	XP_844887	XP_001682341	OQ872521
SR and related proteins	ASF/SF2	NP_008855	No hit			
	SRSF7	NP_0010268 54	OQ397619	XP_827049		
	SRSF3	NP_003008	No hit			
	SRSF9	NP_003760	No hit			
	SRSF10	NP_473357	OQ397620			
	SRSF5	NP_0010345 54	No hit			
	SRSF6	NP_006266	No hit			
	SRSF4	NP_005617	No hit			
	SRSF2	NP_0011823 56	No hit			
	hTra2a	NP_037425	No hit			
	hTra2b/SF RS10	NP_004584	OQ397621			
	ARGLU1	NP_060481	No hit			
hnRNP proteins	hnRNP A0/A1/A3/ A/B/A2/B1	NP_006796<br>NP_112420<br>NP_0013171 78<br>NP_004490<br>NP_002128	OQ397622			
	hnRNP C1/C2	NP_0010709 10	No hit			
	hnRNP D	NP_112738	No hit	XP_846936		
	hnRNP F	NP_0010916 74	No hit	XP_951606§		OQ872522
	hnRNP G	NP_002130	No hit			
	hnRNP H1/2/3	NP_0012442 22<br>NP_0010275 65<br>NP_0013093 67	No hit	XP_951606§		OQ872523<br>OQ872524
	hnRNP K	NP_002131	No hit			

	hnRNP M	NP_005959	OQ397623<br>OQ397624			OQ872525
	hnRNP Q	NP_006363	No hit			
	hnRNP R	NP_005817	OQ397625<br>OQ397602			
	hnRNP U	NP_114032	No hit			
	RALY	NP_057951	No hit			
	E1B-AP5	NP_008971	OQ397626<br>OQ397627			OQ872526
	PTBP1/2	NP_002810<br>NP_001287914	OQ397628<br>OQ397629<br>OQ397630<br>OQ397631<br>OQ397632<br>OQ397633	XP_827198	XP_888628	OQ872527<br>OQ872528
	PCBP1/2	NP_006187<br>NP_005007	No hit			
miscellaneous proteins	CFAP20	NP_037374	OP185700	XP_822473	XP_001682971	OQ872529
	CCAR2[&]	NP_001380926	OP185632<br>OP185633			
	NFAR	NP_001381740	No hit			
	ZNF207	NP_003448	OQ397634			OQ872530
	NRIP2	NP_113662	No hit			
	SAP30BP	NP_037392	No hit			
	DDX9	NP_001348	No hit			
	DNAJC6	NP_055602	No hit			
	PPP1CA	NP_002699	OQ397635<br>OQ397636	XP_844512	XP_001686170<br>XP_001686172<br>XP_001686173	OQ872531<br>OQ872532
	GCFC	NP_057715	No hit			
	BAG2	NP_004273	No hit			
	CIRP	NP_001271	OP185702	XP_845656	XP_001685908	
	NIPP1	NP_054829	OQ397637	XP_845871	XP_001683205	OQ872533
	SMN1	NP_000335	No hit			
	CLNS1A	NP_001284	No hit			
	MEP50	NP_077007	No hit			
	PRMT5	NP_006100	OP185704	XP_822322	XP_001683098	OQ872534
	SEC31B	NP_056305	No hit			
	RBBP6	NP_008841	OP185705	XP_827503	XP_003722699	OQ918097
	AGGF1	NP_060516	No hit			
	CELF1	NP_001363371	OP279271			
	RBFOX2	NP_001076048	No hit			
	Quaking/Sam68/Slm-2[&]	NP_006766<br>NP_006550<br>NP_006549	OP185631			

[&]Homologous gene pairs for U1A/U2B", SRPK1/SRPK2, HSPA8/HSPA1A, SF1/Quaking/Sam68/Sam-2, CCAR1/CCAR2 and p68/p72

[#]Gene accession numbers (Genbank) for *E. gracilis* and *D. papillatum*

[$]Trypanosoma-specific splicing proteins, including several LSms and U5-Cwc21, are not included due to low similarity to their respective human counterparts

[§]Homologous to both hnRNP F and H1/2/3
